# Chemical and pharmacological evaluations on the extract of *Scutellaria baicalensis* Georgi (Huang-Qin) prepared by various extraction methods

**DOI:** 10.1186/s40064-016-3115-3

**Published:** 2016-08-30

**Authors:** Xuelin Zhou, Pou Seng Choi, Jia-Ming Yang, Penelope M. Y. Or, Pui Man Hoi, Simon M. Y. Lee, George P. H. Leung, Sai Ming Ngai, Siu Kai Kong, Ho Pui Ho, Melody Y. M. Wong, Shun Wan Chan, John H. K. Yeung, Yiu Wa Kwan

**Affiliations:** 1 School of Biomedical Sciences, Faculty of Medicine, The Chinese University of Hong Kong, Hong Kong, China; 2 Institute of Chinese Medical Sciences, University of Macau, Macao, China; 3 School of Chinese Medicine, Faculty of Sciences, The Chinese University of Hong Kong, Hong Kong, China; 4 Department of Pharmacology and Pharmacy, The University of Hong Kong, Hong Kong, China; 5 School of Life Sciences, Faculty of Sciences, The Chinese University of Hong Kong, Hong Kong, China; 6 Department of Electronic Engineering, Faculty of Engineering, The Chinese University of Hong Kong, Hong Kong, China; 7 State Key Laboratory of Chinese Medicine and Molecular Pharmacology, Department of Applied Biology and Chemical Technology, The Hong Kong Polytechnic University, Hong Kong, China; 8 Department of Food and Health Sciences, Faculty of Science and Technology, The Technological and Higher Education Institute of Hong Kong, Hong Kong, China

**Keywords:** *Scutellaria baicalensis* Georgi, Extraction efficiency, Antioxidant properties, Herb–drug interaction

## Abstract

**Background:**

This study reported a comprehensive approach (comparing the extraction yields, chemical profiles, antioxidant properties and CYP450-inhibitory effects) to evaluated the effectiveness of various extraction methods [microwave-assisted extraction using water (MAE-W), heat reflux extraction using water (HRE-W), ultrasonic extraction using 70 % ethanol and ultrasonic extraction using ethanol (UE-E)] for Huang-Qin (HQ), the dried root of *Scutellaria baicalensis* Georgi.

**Results:**

The HQ extraction efficiency by MAE-W was the best. The chemical profiles of extracts obtained using HRE-W and MAE-W were similar; whereas more flavones but less flavone glycosides were detected in the UE-E extract. There was no difference in the antioxidant properties among different extracts. In vitro human liver microsome assays illustrated that all extracts possessed herb–drug interaction potentials but the UE-E extract are shown with a potent interaction with CYP3A4-metabolized drugs.

**Conclusion:**

MAE-W is a favorable method for the preparation of HQ extracts based on extraction yield, pharmacological properties and safety.

## Background

There are different widely available and affordable extraction methods such as heat reflux extraction (HRE), microwave-assisted extraction (MAE) and ultrasonic extraction (UE) for routine herbal extraction. Comparisons always focus on the yields of crude extracts of the same herb using different extraction methods, but more importantly comparing the pharmacological/toxicological effects of extracts obtained are lacking. In this study, Huang-Qin (HQ), the root of *Scutellaria baicalensis* Georgi, was chosen as the model to evaluate the effectiveness of different extraction methods because its chemical properties and major components have been well studied and information is available in the literature for comparison. A successful search for a cost-efficient (i.e. a better yield of active component with similar/improved therapeutic activities using the same amount of herb) extraction method for this herb can lay down the foundation for future use of this method for other more expensive herbs with favourable outcomes both financially and therapeutically.

HQ is a widely used medicinal plant for treatment of various diseases (Chien et al. 2010; Shang et al. 2010); it is also used as food supplement for health-care purposes because of its antioxidant properties (Chan et al. 2008). HQ is rich in flavones and flavone glycosides (baicalein, wogonin and their glycosides), while baicalin and wogonoside are generally believed to be its major active ingredients. Flavones and flavone glycosides found in HQ are responsible for its reported anti-cancer, anti-inflammatory (Li-Weber 2009), vasorelaxant (Huang et al. 2005), anti-viral and other biological activities (Chan et al. 2011; Cui et al. 2010; Liu et al. 2011; Zhang et al. 2015). Currently, there are more than 87 formulations and concoctions containing HQ extract indexed in the *Chinese Pharmacopoeia* (Comission 2010). As mentioned in the *Chinese Pharmacopeia*, HQ is routinely prepared using heat reflux extraction using water (HRE-W) procedures and baicalin is used as reference for quality control purpose. In addition, heat reflux extraction using ethanol (HRE-E), ultrasonic extraction (UE) with organic solvents (and water) and microwave-assisted extraction (MAE) (Horvath et al. 2005; You et al. 2010) are alternative extraction methods that in general provide better yields of HQ crude extract. However, simply looking at the yield of extract obtained is bias because extraction methods making use of ethanol/organic solvent in general result in better yields of extract, but with more hydrophobic products. Moreover, as observed in a Danshen (*Salvia Miltiorrhiza*) study, extracts prepared by different extraction methods have varied pharmacological effects (Zhou et al. 2012a). Unfortunately, extraction using organic solvent is not a common household practice, and more importantly unwanted or even toxic substances could accumulate in the extract at levels which are high enough to pose serious health hazards, but may exist only in minimal levels when water is used.

The aim of this study was to quantify and compare the yields of HQ extracts given by different extraction methods, namely conventional heat reflux extraction using water (HRE-W), microwave-assisted extraction using water (MAE-W) and ultrasonic extraction using ethanol (70 % vol/vol) (UE-E) or water (UE-W) as solvent. In addition, the chemical profiles of different HQ extracts were characterized, their anti-oxidant potentials were estimated, and the magnitudes of CYP450s-mediated herb–drug interactions were evaluated.

## Methods

### Reagents and materials

HQ herb was purchased from a TCM distributor (Man-San-Tong, Macao, China) and ground into fine powder for extraction. The voucher sample (Lot. No. 11-803) of HQ herb was authenticated by a botanist, Ms. Huan Zhang, and kept in Prof. Yiu Wa Kwan’s laboratory, School of Biomedical Sciences, Faculty of Medicine, The Chinese University of Hong Kong, Hong Kong. Authentic standards (purity > 95 %) of baicalin and baicalein were purchased from Nanjing Zelang Co. (Nanjing, China); those of wogonoside and wogonin were from Hong Kong Jockey Club Institute of Chinese Medicine (Hong Kong, China). Pooled human liver microsomes (HLMs) were supplied by GenTest Corporation (Woburn, MA, USA). 6-hydroxyflavone, trifluoroacetic acid (TFA, HPLC grade) and other chemicals were purchased from Sigma-Aldrich Chemicals Co. (St. Louis, MO, USA).

### Extraction procedures

In order to make an accurate/meaningful comparison, 50 g of ground HQ was immersed in different solvents using various extraction methods. Heat reflux extraction using water (HRE-W) was performed according to the *Chinese Pharmacopeia* (Comission 2010) and HQ powder was extracted (100 °C, 2 h). For microwave-assisted extraction using water (MAE-W), HQ powder was put into a 500-ml flask inside a MAS-II microwave oven (Shanghai SINEO Microwave Co., China) with microwave power of 300 W connected to a water-jacket condenser to alter the atmospheric pressure inside the flask if necessary and heated at 100 °C for 2 h. For ultrasonic extraction (UE) using water (UE-W) or ethanol (UE-E), HQ powder was extracted (1 h) at 45 °C with either water or ethanol (70 % vol/vol) with the same ultrasonic frequency (40 kHz) and power (250 W) (Branson 8510 ultrasonicator, Branson, USA). All herbal mixtures obtained using different extraction methods were centrifuged at 5000*g* for 20 min before the supernatant of each sample was carefully collected. Supernatant obtained from the UE-E method was condensed under reduced pressure to remove ethanol. All extracts collected were then freeze-dried (Scanlaf Coolsafe 110-4 Freeze-dryer, LaboGene, Denmark) and weighed for extraction yield calculation before they were ground into powder. All ground powders were stored at −80 °C in a light-proof container before use. Three independent extractions were performed, and these three different batches of samples were individually evaluated in the following assays.

### Identification and quantification of chemical profiles

Chemical analysis was carried out using an Agilent 1100 LC/MSD Trap (Agilent, Germany) equipped with a binary pump, an online degasser, an auto-sampler, a Diode Array Detector (DAD) and an APCI source using positive and negative ionization modes. Chromatography was performed at 30 °C with a Grace Alltima C18 column (250 mm × 4.6 mm, 5 μm) protected by a C18 guard column (10 mm × 4.6 mm, 5 μm). The mobile phase constituted (A) 0.05 % TFA water (vol/vol) and (B) acetonitrile in a gradient elution: 20 % B at 0–10 min, 20–55 % B at 15–45 min, 55–80 % B at 45–50 min, and 80 % B at 50–55 min (Horvath et al. 2005). Injection volume was 50 μl, and flow rate was set at 0.7 ml/min with detection wavelength at 280 nm and reference wavelength at 580 nm. The interface and mass spectrum detector (MSD) parameters employed were as follows: nebulizer pressure, 50 psi; dry gas, 5.0 l/min; dry gas temperature, 350 °C; vaporizer temperature: 400 °C; scan range 100–1100 m/z; target mass, 500 m/z; compound stability, 100 %. Stock solutions (10 mg/ml) of all authentic standards were prepared in methanol of analytical grade, and stored at 4 °C before use. Samples of HQ extracts were freshly prepared in water (10 mg/ml). Authentic standards and extract samples were diluted (methanol–water, 50:50, vol/vol) to the desired concentrations. 6-hydroxyflavone was used as the internal standard (I.S.), and 10 μl of I.S. (50 μg/ml) was used with 50 μl of individual sample. The quantitative method used in this study was validated based on the detection of baicalin, baicalein, wogonoside and wogonin.

### Evaluation of antioxidant properties

The free radicals scavenging capacities (SC) were measured and compared using the DPPH assay (Chan et al. 2010). Briefly, 5 µl of individual extract (0.625–10 mg/ml) was mixed with 195 μl of DPPH solution (24 mg/l) in a 96-well plate. The reaction was allowed to proceed in the dark for 30 min before the absorbance of the resultant solution was measured at 515 nm using a Micro-plate Absorbance Reader (Bio-Rad, CA, USA). Ascorbic acid (Sigma-Aldrich Chemicals Co., MO, USA) in distilled water served as the positive control. SC_50_ was estimated as the concentration of sample which scavenges 50 % of free radicals.

The FRAP assay was performed in a 96-well plate as described (Firuzi et al. 2005). Briefly, 5 µl of each sample was mixed with 150 µl of freshly prepared FRAP reagent (250 mM acetate buffer, pH 3.6; 8.3 mM 2,4,6-tripyridyl-*s*-triazine dissolved in 40 mM HCl and 16.7 mM FeCl_3_·6H_2_O solution). After incubation at 37 °C for 30 min, the absorbance of the solution was measured at 593 nm. Different concentrations of Fe^2+^ standard solution were used for the construction of the standard curve, and ascorbic acid was used as the positive control.

### Comparison of the protective effects of HQ extracts on hydrogen peroxide-induced cell death

The protective effects of HQ extracts on hydrogen peroxide (H_2_O_2_)-induced cell death were evaluated using rat heart H9c2 cells (Chan et al. 2010). H9c2 cells were cultured in high glucose DMEM with 10 % FBS, 100 units/ml penicillin and 100 μg/ml streptomycin, and seeded into a 96-well plate at a density of 1.5 × 10^4^ cells per well overnight before they were treated with HQ extracts (62.5–500 μg/ml, diluted from 50 mg/ml stock in DMSO) for 24 h. After treatment, the cells were washed twice with warmed PBS, and then 500 μM H_2_O_2_ diluted in DMEM was added and incubated for 6 h, followed by the addition of 3-(4,5-dimethyl-2-thiazolyl)-2,5-diphenyl-2H-tetrazolium bromide (MTT, 0.5 mg/ml in medium) for 3 h. Crystals formed were dissolved in DMSO and the absorbance of the solution was measured at 570 nm. The DMSO (1 %)-treated (without H_2_O_2_ or HQ extracts) group served as solvent control, whereas the H_2_O_2_-treated (plus 1 % DMSO) group was the positive model for determination of cell death. The protective effects of HQ extracts against H_2_O_2_-induced cell death were estimated and compared based on the calculated EC_50_ values of different HQ extracts.

### Inhibition study on CYP450 isoforms

The inhibitory effects of HQ extracts on the activities of human CYP1A2, CYP2C9, CYP2D6 and CYP3A4 in pooled human liver microsomes (HLMs) were studied using enzymatic reactions with probe substrates (50 μM) as follows: CYP1A2, phenacetin O-deethylation; CYP2C9, tolbutamide 4-hydroxylation; CYP2D6, dextromethorphan O-demethylation; CYP3A4, testosterone 6β-hydroxylation. Incubation, samples preparation and subsequent HPLC analysis were carried out as previously described (Wang et al. 2010; Zhou et al. 2012b). Series of concentration of HQ extracts (1–1000 μg/ml) were prepared and the estimated inhibitory potency was expressed as IC_50_ value. To estimate the potential in vivo CYP enzymes inhibition caused by HQ extracts, the volume per dose index (VDI, liter per dose unit) was employed and VDI was calculated as the clinical dose unit of HQ extract divided by IC_50_ value, as reported (Strandell et al. 2004).

### Statistical analysis

Data were statistically analyzed with one-way ANOVA followed by Bunnett’s Post Hoc using Prism v4.0 (GraphPad Inc., CA) and the results were presented as mean ± SEM. *P* value <0.05 was considered statistically significant.

## Results and discussion

Based on the dry weights i.e. the yields of the HQ crude extracts obtained (Table 1), MAE-W was the most effective extraction method (crude extract: 390.8 ± 16.6 mg/g) whereas HRE-W was less effective (crude extract: 297.4 ± 28.4 mg/g) (i.e. MAE-E versus HRE-W: ~30 % increase). Using the UE method (a widely-used efficient method in a previous quality-control study on HQ) (Li et al. 2009), 327.4 ± 19.4 mg/g of HQ crude extract was obtained when ethanol (70 % vol/vol) was used as solvent, whereas only 275.0 ± 9.0 mg/g of crude extract was obtained when water was used. Our results clearly illustrate that among all the extraction methods employed in this study, MAE-W was the most efficient method for herbal extraction based on the yield of HQ crude extract.

**Table 1:** Summary of the yields of crude extracts (dry weight, mg) obtained from ground Huang-Qin (50 g) using different extraction methods

Extraction methods	Abbreviation	Crude extract (mg/g herb)
Heat reflux extraction using water	HRE-W	297.4 ± 28.4
Microwave-assisted extraction using water	MAE-W	390.8 ± 16.6***
Ultrasonic extraction using ethanol (70 % vol/vol)	UE-E	327.4 ± 19.4
Ultrasonic extraction using water	UE-W	275.0 ± 9.0

Results are expressed as mean ± SEM (n = 3)

*** *P* < 0.001 compared to HRE-W

**Fig. 1:**
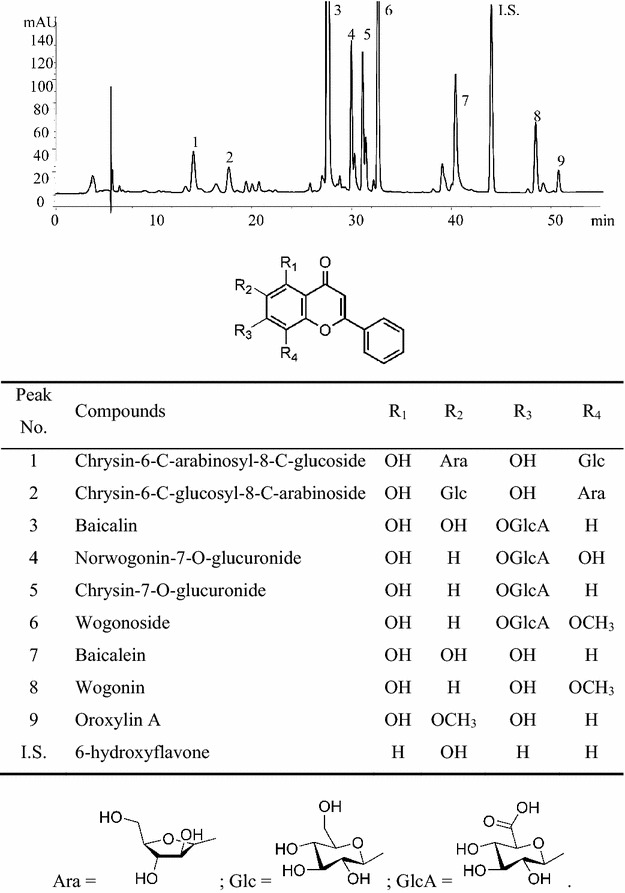
Representative HPLC–UV (at 280 nm) of base peak chromatographic profiles of authentic standards used. Nine major HPLC peaks were detected and identified as flavones. Their chemical structures are also shown. 6-hydroxyflavone was used as the internal standard (I.S.)

Figure 1 illustrates the HPLC–UV (at 280 nm) of base peak chromatographic (BPC) profiles of authentic standards used. Nine major HPLC peaks were detected and identified as flavones in the HPLC chromatogram. Compounds **3**, **6**, **7** and **8** were identified as baicalin, wogonoside, baicalein and wogonin, respectively. Other components were identified using APCI-MS methods (Table 2). The glycoside (e.g. baicalin) exhibited a significant MS signal in both positive and negative modes, whereas aglycone (e.g. baicalein) was observed only in positive mode. In the MS spectra, all analyzed compounds exhibited a noticeable protonated [M + H]^+^ ion in positive mode. However, in negative mode, no consistent pattern was detected. The O-linked glycosides such as baicalin and wogonoside exhibited an intensive adduct ion [M + TFA]^−^ which produced a [M − H]^−^ ion in MS^2^. The multi-stage MS of [M + H]^+^ or [M − H]^−^ of O-linked glycosides provided information about the loss of sugar moiety, and the structure of aglycones was formed. Compounds **4** and **5** were O-linked glycosides, and they were identified as norwogonin-7-O-glucuronide (norwogonin-7-O-GlcA) and chrysin-7-O-glucuronide (chrysin-7-O-GlcA) respectively as previously reported (Han et al. 2007; Liu et al. 2009). On the other hand, Compounds **1** and **2** demonstrated a dominant pseudo-molecular ion [M − H]^−^ in their respective MS spectra, illustrating the existence of [M − H − 60]^−^, [M − H − 90]^−^ and [M − H − 120]^−^ ions, which are the characteristics of typical C-linked glycosides (Han et al. 2007; Liu et al. 2009). Moreover, the MS^n^ data suggested that Compound **1** was chrysin-6-C-arabinosyl-8-C-glucoside (chrysin-6-C-Ara-8-C-Glc) and Compound **2** was chrysin-6-C-glucosyl-8-C-arabinoside (chrysin-6-C-Glc-8-C-Ara). Compound **9** was an isomer of Compound **8** (wogonin) and it was probably oroxylin A according to the retention time and MS fragments.

**Table 2:** Characterization of major ingredients in Huang-Qin extract by HPLC–DAD–APCI–MS

Peak no.^a^	*t*_*R*_ (min)	UV λ_max_	MS^n^ (*m/z*) and relative abundance (% base peak)	Identification
1	14.0	215, 275, 318	APCI^+^—MS: 549(100); MS^2^ [549]: 531(70), 513(60), 495(90), 483(20), 465(45), 429(10), 411(100), 393(30), 363(10); MS^3^ [411]: 393(100), 375(60), 363(70), 333(80), 309(30), 279(28) APCI^−^—MS: 547(100); MS^2^ [547]: 529(20), 511(10), 487(85), 457(100), 427(42), 409(10), 367(60), 337(70); MS^3^ [457]: 367(10), 337(100)	Chrysin-6-C-arabinosyl-8-C-glucoside^c^
2	17.6	215, 275, 318	APCI^+^—MS: 549(100); MS^2^ [549]: 531(100), 513(30), 495(20), 483(45), 465(20), 453(18), 441(18), 411(10), 393(8), 381(6); MS^3^ [531]: 513(80), 495(60), 483(100), 465(75), 453(70), 441(60), 411(12), 393(30), 381(50), 363(40) APCI^−^—MS: 547(100); MS^2^ [547]: 529(15), 487(15), 457(70), 427(100), 367(20), 337(30); MS^3^ [427]: 367(15), 337(100)	Chrysin-6-C-glucosyl-8-C-arabinoside^c^
3	27.3	215, 277, 316	APCI^+^—MS: 447(100), 271(20); MS^2^ [447]: 271(100); MS^3^ [271]: 253(40), 241(100) APCI^−^—MS: 559(100); MS^2^ [559]: 445(100); MS^3^ [445]: 269(100)	Baicalin^b^
4	29.7	215, 280, 360	APCI^+^—MS: 447(100), 271(20); MS^2^ [447]: 271(100) APCI^−^—MS: 559(20), 445(100); MS^2^ [559]: 445(100); MS^3^ [445]: 269(100)	Norwogonin-7-O-glucuronide^c^
5	30.8	215, 273, 312	APCI^+^ – MS: 431(100), 255(20); MS^2^ [431]: 255(100) APCI^−^—MS: 543(100); MS^2^ [543]: 429(100); MS^3^ [429]: 253(100), 173(40)	Chrysin-7-O-glucuronide^c^
6	32.3	215, 275, 349	APCI^+^—MS: 461(100); MS^2^ [461]: 285(100); MS^3^ [285]: 270(100) APCI^−^—MS: 573(100); MS^2^ [573]: 459(100); MS^3^ [459]: 283(100), 268(20), 174(30)	Wogonoside^b^
7	40.1	215, 275, 322	APCI^+^—MS: 271(100); MS^2^ [271]: 241(100)	Baicalein^b^
8	48.0	210, 275	APCI^+^—MS: 285(100); MS^2^ [285]: 270(100)	Wogonin^b^
9	50.5	215, 273, 318	APCI^+^—MS: 285(100); MS^2^ [285]: 270(100)	Oroxylin A^c^

^a^The peak number is the same as indicated in Fig. 1

^b^Identities of compounds were established by comparison with the authentic compounds

^c^On-line identification by HPLC–MS^n^ analysis

**Fig. 2:**
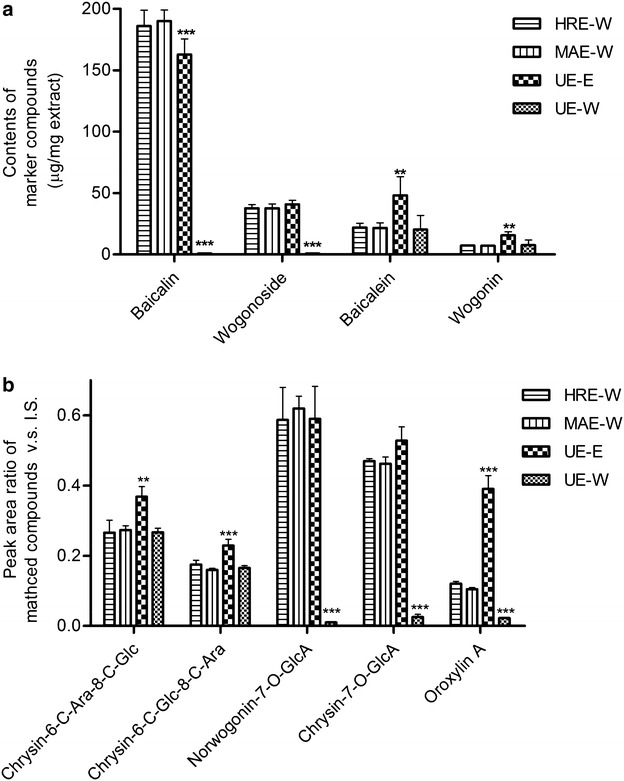
Effects of extraction methods on **a** the contents of marker compounds and **b** the estimated contents of other identified ingredients in Huang-Qin extracts. Results are expressed as mean ± SEM (n = 3). **P < 0.01 and ***P < 0.001 compared to HRE-W

The chemical profiles and quantity of four marker compounds in HQ extracts obtained are shown in Fig. 2a. Our results demonstrated that MAE-W (baicalin, 190.1 ± 4.4 μg/mg; wogonoside, 37.7 ± 1.7 μg/mg; baicalein, 21.8 ± 2.1 μg/mg; wogonin, 7.1 ± 0.2 μg/mg) and HRE-W (baicalin, 186.0 ± 6.4 μg/mg; wogonoside, 37.8 ± 1.5 μg/mg; baicalein, 22.0 ± 1.8 μg/mg; wogonin, 7.3 ± 0.1 μg/mg) methods gave similar results in terms of the quantity of these compounds. In addition, these two extraction methods did not affected the chemical profiles of the HQ extracts obtained with a constant ratio of 5:1 for baicalin/wogonoside. On the other hand, HQ extract contained less baicalin (163.0 ± 6.3 μg/mg) but more baicalein (48.2 ± 7.6 μg/mg) and wogonin (15.6 ± 1.5 μg/mg) when UE-E was used. These results are consistent with the observations of some previous studies on HQ extraction using organic solvent (Horvath et al. 2005; Lin et al. 1996). However, when water was used as solvent in the UE procedures, HQ extract obtained had the least amount of baicalin (0.8 ± 0.3 μg/mg) and wogonoside (0.8 ± 0.3 μg/mg) among all extraction methods used in this study.

Besides, there were no significant differences in the amount of water-soluble components (e.g. chrysin-6-C-Ara-8-C-Glc, chrysin-6-C-Glc-8-C-Ara, norwogonin-7- O-GlcA and chrysin-7-O-GlcA) and lipid components (like oroxylin A) present in the extracts prepared by MAE-W and HRE-W (Fig. 2b). As expected, using UE with ethanol, the yield of most water-soluble components were lower compared with those prepared by MAE-W and HRE-W (e.g. 19 % decrease of baicalin), whereas higher yields of the major lipid-soluble components (e.g. baicalein, 119 %; wogonin, 79 %; oroxylin A, 197 %) were observed probably due to the higher solubility of these compounds in ethanol. When water was used as solvent in UE, the least amount of these identified compounds could be obtained. These results therefore suggested that based on the profiles and quantity of all chemicals detected in the extracts, the MAE-W method is as good as the commonly used HRW-E method, whereas the efficiency and effectiveness of the UE method is highly dependent on the solvent used.

Baicalein, wogonin and their glycosides contribute significantly to the total weight of HQ (Su et al. 2008), and these flavones are strong antioxidants. It is therefore feasible to utilize both the chemical profiles and the antioxidant properties of HQ extract for quality control purposes for all HQ-containing products in the market (Boyle et al. 2011). Table 3 illustrates that there were no significant differences in the free radicals scavenging activities (as estimated by DPPH assay) of extracts prepared by the extraction methods (SC_50_: MAE-W, 1.046 ± 0.064 mg/ml; HRE-W, 0.958 ± 0.032 mg/ml; UE-E, 0.988 ± 0.057 mg/ml). However, extract prepared by the UE-W method has the weakest scavenging activity (SC_50_: 3.899 ± 0.175 mg/ml), whereas ascorbic acid is the strongest scavenger (SC_50_: 0.107 ± 0.004 mg/ml). The results obtained from the FRAP assay demonstrated a similar trend of antioxidant properties of HQ extracts prepared by different extraction methods (Table 3).

**Table 3:** Summary of the antioxidant potential of Huang-Qin extracts prepared by different extraction methods as estimated by DPPH assay (SC_50_, mg/ml) and FRAP assay (μmol Fe^2+^/mg extract)

Samples	DPPH assay (SC_50_, mg/ml)	FRAP assay (μmol Fe^2+^/mg extract)
Ascorbic acid^a^	0.107 ± 0.004***	2278 ± 15***
HRE-W	0.958 ± 0.032	1249 ± 131
MAE-W	1.046 ± 0.064	1306 ± 71
UE-E	0.988 ± 0.057	1359 ± 29
UE-W	3.899 ± 0.175***	541.6 ± 98***

Results are expressed as mean ± SEM (n = 3)

*** *P* < 0.001 compared to HRE-W

^a^1 mM was used

Due to the relatively low yield (the lowest yield among all extraction methods) of bioactive components and antioxidant activity of the extract obtained, water extract of HQ obtained from UE was therefore not evaluated. As illustrated in Table 4, all HQ extracts (HRWE, MAE-W and UE-E) demonstrated a similar protective effect (EC_50_ of 176.4 ± 14.5, 190.6 ± 12.1 and 165.7 ± 19.9 μg/ml, respectively) against H_2_O_2_-induced cell death as determined by MTT assay. Interestingly, these cell-based results were comparable to the results of antioxidant/free radicals scavenging assays as discussed in previous sections.

**Table 4:** Summary of the protective effects [expressed as EC_50_ (μg/ml)] of Huang-Qin extracts prepared by different extraction methods against H_2_O_2_-induced cell death (estimated by MTT assay) using rat heart H9c2 cells

Samples	EC_50_ (μg/ml)
HRE-W	176.4 ± 14.5
MAE-W	190.6 ± 12.1
UE-E	165.7 ± 19.9

Results are expressed as mean ± SEM (n = 3)

There is little information in the literature that compares the hepatic enzymes inhibitory effects of HQ extracts prepared by different methods. Some HQ flavones, which are abundant especially when using ethanol as solvent for extraction, demonstrated substantial inhibition of human hepatic drug metabolizing enzymes cytochrome P450s (CYP 450s) such as CYP2C9 (Si et al. 2009), CYP3A4, CYP1A2 (Kim et al. 2002; Li et al. 2011) and CYP2D6 (Mo et al. 2012). Therefore, it is important to evaluate whether the extract obtained using MAE-W method has different inhibitory profiles compared with other extraction methods on hepatic CYP450 enzymes. HQ extract obtained by the HRE-W method was used as reference for comparison.

Extracts obtained by MAE-W demonstrated a moderate inhibition of CYP1A2 and CYP2C9 (as indicated by 9 and 20 % decrease in IC_50_ values, respectively) whereas an “enhancement” rather than an inhibition (i.e. ~ 40 % increase in IC_50_ value) of CYP3A4 activity was observed (Table 5). There was a similar magnitude of CYP2D6 inhibition caused by HQ extracts obtained by MAE-W and HRE-W. On the other hand, extract obtained by UE-E had a marked inhibition on CYP1A2, CYP2C9 and CYP2D6 (as illustrated by 63 to 71 % decrease in IC_50_ values) and only a weak inhibition (~39 % decrease in IC_50_) of CYP3A4 was observed. Previous studies reported that HQ extract caused CYP450s inhibition mainly through the flavones and flavones glycosides present in the extract (Kim et al. 2002; Li et al. 2011). However, CYP1A2 inhibition by flavone glycosides of HQ extracts has not been reported.

**Table 5:** Summary of the inhibitory effects [expressed as IC_50_ (μg/ml) and volume per dose index (VDI, L/dose unit)] of Huang-Qin (HQ) extracts prepared by different extraction methods on model probes’ metabolism

Samples	CYP1A2		CYP2C9		CYP2D6		CYP3A4	
	IC_50_	VDI	IC_50_	VDI	IC_50_	VDI	IC_50_	VDI
HRE-W	8.13 ± 0.08	80.0	38.87 ± 0.60	16.7	131.3 ± 5.35	4.95	174.1 ± 4.86	3.74
MAE-W	7.42 ± 0.20*	87.6	30.71 ± 0.84***	21.2	135.3 ± 6.43	4.80	243.0 ± 11.81**	2.68
UE-E	3.00 ± 0.12***	216.7	11.25 ± 0.66***	57.8	38.29 ± 1.28***	17.0	106.6 ± 4.53**	6.10
Positive controls^a^	0.640	–	0.138	–	0.292	–	0.047	–

Results are expressed as mean ± SEM (n = 3)

* *P* < 0.05; ** *P* < 0.01 and *** *P* < 0.001 compared to HRE-W

^a^Furafylline for CYP1A2; Sulfaphenazole for CYP2C9; Quinidine for CYP2D6; Ketoconazole for CYP3A4. Results of the positive controls included in this Table for comparison have been published in our previous work (Wang et al. 2010; Zhou et al. 2012b)

Volume per dose index (VDI) has been employed for the estimation of possible in vivo herb–drug interactions in in vitro incubation using human pooled liver microsomes. As proposed by Strandell et al. (2004), when VDI is greater than 4 l (i.e. human blood volume), it is likely that in vivo herb–drug interactions occurred with the participation of hepatic CYP enzymes. According to the National Drug Standards (Traditional Chinese Medicine Prescription, Volume 2) (Ministry of Health, PR of China), the dose unit of HQ tablets is about 650 mg HQ extract in which 117 mg is baicalin. Based on this dose unit and the respective IC_50_ values, the VDI of individual CYP450 isoforms were estimated (Table 5). The estimated VDIs of all HQ extracts examined on CYP1A2, CYP2C9 and CYP2D6 enzymes were greater than 4 l per dose unit. More importantly, based on the estimated VDIs, only the extract obtained using UE-E method illustrated a significant in vivo herb–drug interaction with CYP3A4-metabolized Western drugs. Taken together, our results clearly demonstrated that HQ extracts, irrespective of the extraction methods/solvents used, possess significant herb–drug interactions with CYPs-metabolized Western medicines.

Recent pharmacological and toxicological studies indicated that water-soluble components of HQ extract exhibit a variety of therapeutically useful activities with relatively low toxicity (Yimam et al. 2010), whereas long-term toxicity (e.g. damage to the bile duct) of extracts prepared using organic solvent has been reported (Ong et al. 2004) although herbal extraction using organic solvent (e.g. UE-E in this study which possesses a significant herb–drug interaction with CYP3A4-metabolized drugs) is not a common practice for human consumption. Taken together, our results illustrate that MAE-W provided an effective and efficient procedure in terms of a higher yield of crude extract collected than the conventional HRE-W, and more importantly HQ extract prepared by this procedure has similar pharmacological activities with a lesser magnitude of herb–drug interactions when compared with extracts prepared using other organic-solvent extraction methods. It remains to be determined whether this favorable cost-efficient extraction method could be used in preparation of extracts of more expensive Chinese herb such as Cordyceps for therapeutic uses without affecting their pharmacological effects.

## References

[CR1] Boyle SP, Doolan PJ, Andrews CE, Reid RG (2011). Evaluation of quality control strategies in *Scutellaria* herbal medicines. J Pharm Biomed Anal.

[CR2] Chan SW, Li S, Kwok CY, Benzie IFF, Szeto YT, Guo DJ, He XP, Yu PHF (2008). Antioxidant activity of Chinese medicinal herbs. Pharm Biol.

[CR3] Chan E, Wong CY, Wan CW, Kwok CY, Wu JH, Ng KM, So CH, Au AL, Poon CC, Seto SW, Kwan YW, Yu PH, Chan SW (2010). Evaluation of anti-oxidant capacity of root of *Scutellaria baicalensis* Georgi, in comparison with roots of *Polygonum multiflorum* Thunb and *Panax ginseng* CA Meyer. Am J Chin Med.

[CR4] Chan E, Liu XX, Guo DJ, Kwan YW, Leung GP, Lee SM, Chan SW (2011). Extract of *Scutellaria baicalensis* Georgi root exerts protection against myocardial ischemia-reperfusion injury in rats. Am J Chin Med.

[CR5] Chien CF, Wu YT, Tsai TH (2010). Biological analysis of herbal medicines used for the treatment of liver diseases. Biomed Chromatogr.

[CR6] Comission CP (2010). Chinese Pharmacopoeia version 2010.

[CR7] Cui X, Wang Y, Kokudo N, Fang D, Tang W (2010). Traditional Chinese medicine and related active compounds against hepatitis B virus infection. Biosci Trends.

[CR8] Firuzi O, Lacanna A, Petrucci R, Marrosu G, Saso L (2005). Evaluation of the antioxidant activity of flavonoids by “ferric reducing antioxidant power” assay and cyclic voltammetry. Biochim Biophys Acta.

[CR9] Han J, Ye M, Xu M, Sun J, Wang B, Guo D (2007). Characterization of flavonoids in the traditional Chinese herbal medicine-Huangqin by liquid chromatography coupled with electrospray ionization mass spectrometry. J Chromatogr B.

[CR10] Horvath CR, Martos PA, Saxena PK (2005). Identification and quantification of eight flavones in root and shoot tissues of the medicinal plant Huang-Qin (*Scutellaria baicalensis* Georgi) using high-performance liquid chromatography with diode array and mass spectrometric detection. J Chromatogr A.

[CR11] Huang Y, Tsang SY, Yao X, Chen ZY (2005). Biological properties of baicalein in cardiovascular system. Curr Drug Targets Cardiovasc Haematol Disord.

[CR12] Kim JY, Lee S, Kim DH, Kim BR, Park R, Lee BM (2002). Effects of flavonoids isolated from *Scutellariae radix* on cytochrome P-450 activities in human liver microsomes. J Toxicol Environ Health A.

[CR13] Li C, Zhou L, Lin G, Zuo Z (2009). Contents of major bioactive flavones in proprietary traditional Chinese medicine products and reference herb of *Radix Scutellariae*. J Pharm Biomed Anal.

[CR14] Li T, Li N, Guo Q, Ji H, Zhao D, Xie S, Li X, Qiu Z, Han D, Chen X, You Q (2011). Inhibitory effects of Wogonin on catalytic activity of cytochrome P450 enzyme in human liver microsomes. Eur J Drug Metab Pharmacokinet.

[CR15] Lin SJ, Tseng HH, Wen KC, Suen TT (1996). Determination of gentiopicroside, mangiferin, palmatine, berberine, baicalin, wogonin and glycyrrhizin in the traditional Chinese medicinal preparation Sann-Joong-Kuey-Jian-Tang by high-performance liquid chromatography. J Chromatogr A.

[CR16] Liu G, Ma J, Chen Y, Tian Q, Shen Y, Wang X, Chen B, Yao S (2009). Investigation of flavonoid profile of *Scutellaria bacalensis* Georgi by high performance liquid chromatography with diode array detection and electrospray ion trap mass spectrometry. J Chromatogr A.

[CR17] Liu G, Rajesh N, Wang X, Zhang M, Wu Q, Li S, Chen B, Yao S (2011). Identification of flavonoids in the stems and leaves of *Scutellaria baicalensis* Georgi. J Chromatogr B.

[CR18] Li-Weber M (2009). New therapeutic aspects of flavones: the anticancer properties of *Scutellaria* and its main active constituents Wogonin, Baicalein and Baicalin. Cancer Treat Rev.

[CR19] Mo SL, Liu WF, Chen Y, Luo HB, Sun LB, Chen XW, Zhou ZW, Sneed KB, Li CG, Du YM, Liang J, Zhou SF (2012). Ligand- and protein-based modeling studies of the inhibitors of human cytochrome P450 2D6 and a virtual screening for potential inhibitors from the Chinese herbal medicine, Scutellaria baicalensis (Huangqin, Baikal Skullcap). Comb Chem High Throughput Screen.

[CR20] Ong ES, Len SM, Lee AC, Chui P, Chooi KF (2004). Proteomic analysis of mouse liver for the evaluation of effects of *Scutellariae radix* by liquid chromatography with tandem mass spectrometry. Rapid Commun Mass Spectrom.

[CR21] Shang X, He X, Li M, Zhang R, Fan P, Zhang Q, Jia Z (2010). The genus *Scutellaria* an ethnopharmacological and phytochemical review. J Ethnopharmacol.

[CR22] Si D, Wang Y, Zhou YH, Guo Y, Wang J, Zhou H, Li ZS, Fawcett JP (2009). Mechanism of CYP2C9 inhibition by flavones and flavonols. Drug Metab Dispos.

[CR23] Strandell J, Neil A, Carlin G (2004). An approach to the in vitro evaluation of potential for cytochrome P450 enzyme inhibition from herbals and other natural remedies. Phytomedicine.

[CR24] Su S, He CM, Li LC, Chen JK, Zhou TS (2008). Genetic characterization and phytochemical analysis of wild and cultivated populations of *Scutellaria baicalensis*. Chem Biodivers.

[CR25] Wang X, Cheung CM, Lee WY, Or PM, Yeung JH (2010). Major tanshinones of Danshen (*Salvia miltiorrhiza*) exhibit different modes of inhibition on human CYP1A2, CYP2C9, CYP2E1 and CYP3A4 activities in vitro. Phytomedicine.

[CR26] Yimam M, Zhao Y, Ma W, Jia Q, Do SG, Shin JH (2010). 90-day oral toxicity study of UP446, a combination of defined extracts of *Scutellaria baicalensis* and *Acacia catechu*, in rats. Food Chem Toxicol.

[CR27] You J, Gao S, Jin H, Li W, Zhang H, Yu A (2010). On-line continuous flow ultrasonic extraction coupled with high performance liquid chromatographic separation for determination of the flavonoids from root of *Scutellaria baicalensis* Georgi. J Chromatogr A.

[CR28] Zhang SF, Dong YC, Zhang XF, Wu XG, Cheng JJ, Guan LH, Shang YZ (2015). Flavonoids from *Scutellaria* attenuate okadaic acid-induced neuronal damage in rats. Brain Injury.

[CR29] Zhou X, Chan SW, Tseng HL, Deng Y, Hoi PM, Choi PS, Or PM, Yang JM, Lam FF, Lee SM, Leung GP, Kong SK, Ho HP, Kwan YW, Yeung JH (2012). Danshensu is the major marker for the antioxidant and vasorelaxation effects of Danshen (*Salvia miltiorrhiza*) water-extracts produced by different heat water-extractions. Phytomedicine.

[CR30] Zhou X, Wang Y, Or PM, Wan DC, Kwan YW, Yeung JH (2012). Molecular docking and enzyme kinetic studies of dihydrotanshinone on metabolism of a model CYP2D6 probe substrate in human liver microsomes. Phytomedicine.

